# Applying a new method for studying the functional state of hemostasis in clinical practice

**DOI:** 10.1186/cc9857

**Published:** 2011-03-11

**Authors:** O Tarabrin, I Tyutrin, S Kalinchuk, A Turenko, S Shcherbakov, D Gavrychenko

**Affiliations:** 1 Odessa Medical University, Odessa, Ukraine; 2 Siberian State Medical University, Tomsk, Russia; 3 Odessa Regional Clinical Hospital, Odessa, Ukraine

## Introduction

It is known that deep vein thrombosis of lower extremities and pulmonary embolism occupies an important place in the structure of postoperative morbidity and mortality.

## Methods

After ethics approval and informed consent, we studied the functional state of hemostasis in a group of 40 healthy volunteers who were not receiving drugs affecting coagulation and in 37 patients with postphlebothrombotic syndrome (PPTS). In patients with PPTS we conducted baseline studies of coagulation state and daily monitoring of dynamic changes in the functional state of hemostasis, a comparative evaluation of performance low-frequency piezoelectric vibration hemoviscoelastography (LPVH) and the platelet aggregation test (PAT), standard coagulation tests (SCT), and thromboelastogram (TEG).

## Results

It was found that the LPVH correlated with SCT, PAT and TEG. However, our proposed method is more voluminous: indexes ICC (the intensity of the contact phase of coagulation), t1 (the time the contact phase of coagulation), and A0 (initial rate of aggregation of blood) were consistent PAT indexes; indexes ICD (the intensity of coagulation drive), CTA (a constant thrombin activity) and CIP (the clot intensity of the polymerization) for SCT and TEG. In addition, the advantage of this method is to determine the intensity of fibrinolysis - with indicator IRLS (the intensity of the retraction and clot lysis).

## Conclusions

LPVH allows one to make the total assessment of all parts of hemostasis: from initial viscosity and platelet aggregation to coagulation and lysis of clots, as well as their interaction. These data are objective and informative, as evidenced by close correlation with the performance of traditional coagulation methods.

